# Building a bioelectronic medicine movement 2019: insights from leaders in industry, academia, and research

**DOI:** 10.1186/s42234-020-0037-8

**Published:** 2020-01-31

**Authors:** Alliance for Advancing Bioelectronic Medicine, Samuel Asirvatham, Ken Londoner, Murali Aravamudan, Thomas Deering, Hein Heidbuchel, Suraj Kapa, Barry Keenan, Elad Maor, Soeren Mattke, Lefkos T. Middleton, Valentin Pavlov, Douglas Weber

**Affiliations:** BioSig Technologies, Inc., Westport, CT USA

**Keywords:** Bioelectronics, Bioelectronic medicine, Bioelectronic therapies, Collaboration, Definition, Narrative, Engagement, Messaging, Policy, Stakeholders, Regulators, Investors, Payors

## Abstract

In April 2019, a select group of medical, academic, and private-sector leaders in bioelectronic medicine convened in Geneva to discuss the potential for building a cross-disciplinary movement that would advance the field with key stakeholders – both those who are already active in research and commercialization as well as those who will influence the pace of development and uptake of innovative technologies and treatments. Hosted by BioSig Technologies and physicians from the Mayo Clinic, the roundtable was unique in its focus on what it will take to advance awareness of bioelectronic medicine, including a shared definition, unified narrative, and set of tailored messages to win over key audiences. The attendees developed a consensus on these issues and agreed to form a working group beyond the roundtable, which has since evolved into the Alliance for Advancing Bioelectronic Medicine. This meeting report summarizes the key insights from the roundtable, including a call to action aimed at accelerating growth and collaboration across the field.

## Introduction

Bioelectronic medicine is one of the most innovative and exciting fields in healthcare, with the potential to advance diagnosis and care for people suffering from a range of diseases. These technologies use targeted electrical signals to diagnose and treat diseases, which can provide important advantages for a variety of patients and help to address areas of unmet need.

Bioelectronic medicine is already used to diagnose and treat a variety of challenging diseases, such as arrhythmias, Parkinson’s, major depressive disorder, treatment-resistant epilepsy, incontinence, and chronic pain. In recent years, researchers and companies have continued to expand the field’s applications, preparing a wave of new innovations to enter the market. For example, preliminary research suggests that bioelectronic medicine can detect “silent,” asymptomatic heart conditions, regulate the body’s metabolic and inflammatory control systems, and provide more accessible, affordable approaches to global health. As a result, the field has entered a critical stage in its development, on the cusp of key advances and greater adoption.

However, bioelectronic medicine stakeholders – including companies, research institutes, universities, private investors, public funders, and others – will need to address significant challenges to realize the field’s full potential. Currently, these stakeholders are fragmented, with relatively little collaboration on shared issues or a single public narrative. Outside the field, awareness and support remains limited among key audiences, including patients, providers, policymakers and regulators, payers, and others. As a result, current policy, regulation, and investment do not fully support the field’s growth.

In April 2019, a select group of medical, academic, and private-sector leaders gathered in Geneva, Switzerland to discuss these issues, explore shared goals, and collaborate on new approaches – with an eye to building a powerful global movement around bioelectronic medicine. Co-hosted by BioSig Technologies and physicians from the Mayo Clinic, and facilitated by High Lantern Group, this first-of-its-kind roundtable brought together prominent experts to discuss the current state of the field, future opportunities and challenges, and next steps toward harnessing the full potential of bioelectronic medicine.

This report summarizes insights from the roundtable in three sections:

- **The transformative potential of bioelectronic medicine:** briefly sketches the bioelectronic medicine landscape and its potential to advance healthcare through earlier and less costly diagnosis and treatment of a broad range of diseases.
- **Defining a shared vision for bioelectronic medicine:** proposes a shared definition, unified narrative, and set of tailored messages to win over key audiences. We see these as missing pieces in the field today, essential catalysts needed to build support among the many relevant stakeholder groups – both those who are active within the field, as well as those who will influence the pace of its development. For the field to grow, it must engage each audience with tailored messages that articulate relevant benefits, address their specific concerns, and lead to changes in healthcare policy and practice.
- **Building a bioelectronic medicine movement:** calls on diverse stakeholders to take action to accelerate the development and uptake of bioelectronic medicine technologies and treatments – a potential game-changer for healthcare systems, providers, and patients.

The 2019 roundtable was a crucial first step to ensure leading voices in the field are aligned on key goals, such as promoting broader awareness and support, mobilizing diverse communities, shaping health policy, and sustaining momentum to launch a new movement. To that end, we have created the Alliance for Advancing Bioelectronic Medicine, and we look forward to continued collaboration to achieve these goals through this new group.

## The transformative potential of bioelectronic medicine

Bioelectronic medicine is a rapidly growing field of healthcare that explores how targeted electrical signals can harness the body’s natural mechanisms to diagnose and treat a range of diseases. The field represents not just a narrow category of medical devices, but an entire approach to detecting and treating disease – using electrical pulses and the body’s own mechanisms as an adjunct or alternative to drugs and medical procedures.

The scientific foundations of bioelectronic medicine have been well established in previous publications (Arnold 2018; Famm et al. 2013; Pavlov and Tracey 2019; Zhirnov 2019). To summarize, advances in neuroscience, signal processing, molecular biology, electronics, medical devices, and other fields have converged to enable new approaches and technologies for diagnosing and treating disease. By capturing and analyzing a person’s bioelectronic signals, these technologies can detect disease and, for therapeutic devices, intervene to address the underlying causes.

As shown in Table 1, researchers have identified a very broad range of potential applications, including arrythmias, auto-immune diseases, neurological conditions, diabetes, arthritis, hypertension, pain management, cancer, and others.

**Table 1:** Subfields of Bioelectronic Medicine

Subfields of Bioelectronic Medicine		
Subfield	Description	Examples of Diseases Treated
Cardiac Rhythm Management		
Cardiac Rhythm Management	Pacemakers and other devices to monitor and regulate heart rhythm	Bradycardia, tachycardia, heart failure
Electrophysiology	Catheter-based treatments for cardiac arrythmias	Atrial fibrillation, ventricular tachycardia
Cochlear and Retinal Implants		
Cochlear Implants	Implants that capture, digitize, and transmit sound	Hearing loss/impairment
Retinal Implants	Implants that capture, digitize, and transmit visual information	Retinal degenerative diseases
Central Nervous System (CNS)		
Spinal Cord Stimulation	Devices that stimulate specific nerve fibers in the spinal cord	Chronic pain
Deep Brain Stimulation	Devices that stimulate regions in the brain to address neurological conditions	Treatment-resistant epilepsy, Parkinson’s, major depressive disorder, obsessive compulsive disorder, PTSD
Peripheral Nervous System (PNS)		
Vagus Nerve Stimulation	Devices that stimulate specific areas of the vagus nerve – the body’s “neural highway” – to address a range of conditions	Rheumatoid arthritis, migraine, inflammatory bowel disease, obesity, heart failure
Sacral Nerve Stimulation	Devices that stimulate the sacral nerve to manipulate the bladder or sphincter	Overactive bladder, urinary incontinence

The most well-established subfield within bioelectronic medicine is cardiac rhythm management, which includes pacemakers and other devices that monitor and regulate heart rhythm. Electrophysiology is a related field focusing on catheter-based approaches. Other long-standing subfields include cochlear and retinal implants.

Neuromodulation is a relatively newer but fast-growing area that uses electrical pulses to alter the activity of nerves. It comprises applications in the central nervous system (CNS), including spinal cord stimulation and deep brain stimulation, and applications in the peripheral nervous system (PNS), including vagus nerve stimulation, sacral nerve stimulation, and others.

As the field advances, a key turning point is the emergence of a new generation of technologies that both sense and intervene, continuously and in real time. These “closed- loop” systems monitor a person’s bioelectronic signals and adjust treatment in response to changes in external conditions, internal states, or the effects of the treatment. For example, an artificial pancreas monitors and responds to changes in glucose levels, and certain epilepsy treatments can monitor and respond to brain activity (U.S. Food and Drug Administration 2018; Tsao 2018). In the future, this approach could improve treatment across a number of disease areas, such as depression therapies that monitor and respond to specific neurotransmitters, or inflammatory disease therapies that respond to cytokines (Tsao 2018).

### A fast-growing field

**Fig. 1:**
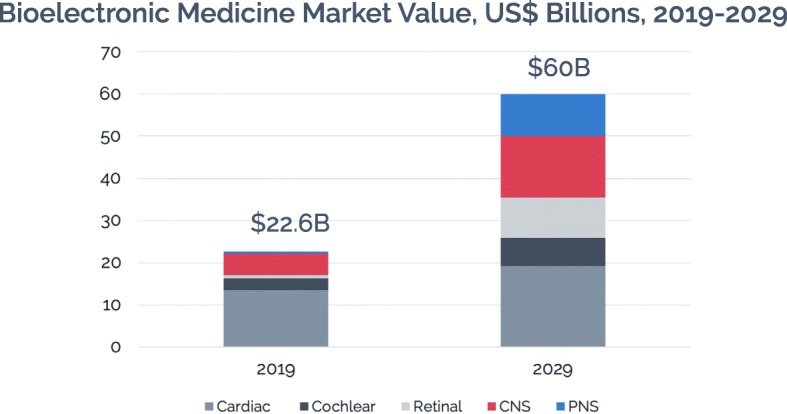
Bioelectronic Medicine Market Value

The market for bioelectronic medicine devices is already large and projected to grow at a rapid pace in the coming years. Figure 1 shows that, according to IDTechEx, bioelectronic medicine is a $22.6 billion market today, projected to reach more than $60 billion in 2029 based on a compound annual growth rate of more than 10% (Tsao 2018). Across the five subfields, the markets for retinal implants and PNS stimulation are forecast to grow much faster than those for CNS stimulation, cardiac solutions, and cochlear implants.

As these figures suggest, bioelectronic medicine has entered a critical period. While some applications of bioelectronic medicine, such as cardiac implantable devices, have been used for decades, a range of new treatments are showing positive trial results, entering the market, and addressing unmet needs among certain patient populations.

Consider the recent IPOs of Inspire Medical in the sleep apnea space, Neuronetics in the psychiatric disorders space, and electroCore in the neurologic and metabolic disorders space (Renaissance Capital 2018; Hale 2018; Mathias 2018). These examples demonstrate that bioelectronic medicine companies can find a path to commercial viability and real-world impact. Dozens of early-stage companies are now working towards this goal, with applications across the spectrum of bioelectronic medicine.

### Current challenges and future perspectives

Looking ahead we see a range of opportunities where bioelectronic medicine could provide significant health, societal, and economic value. These speak to the long-term potential of the field.

It should be noted that many of these applications are still in the pre-clinical, experimental, or theoretical stage, and the field will need to overcome today’s significant challenges to achieve widespread, real-world impact. In particular, there is a need for greater understanding and evidence of the basic science and fundamental mechanisms of these applications. High-quality, validated evidence of health and cost benefits, as well as the risk of adverse effects, will also be needed to win broad acceptance and adoption.

However, early success stories and emerging applications indicate what could be possible as the field continues to grow, develop, and prove its value – pointing to a broad range of opportunities to drive meaningful progress.

#### Treatment advances for areas of unmet need

Bioelectronic medicine could supplement therapeutic approaches to deliver advances for diseases with no available treatments currently or where existing treatments are not highly effective or cause severe side effects. Many of the applications now entering the market have targeted these disease areas, such as treatment-resistant epilepsy, migraine, major depressive disorder, and sleep apnea.

Neuropsychiatric disorders represent a particularly pressing area of need. Conditions like major depressive disorder, treatment-resistant epilepsy, and Parkinson’s affect millions of people, but conventional approaches have limited efficacy for significant shares of the patient population. For example, up to one-third of people with major depression don’t achieve remission with current treatment options (Al-Harbi 2012), and 30–40% of people with epilepsy don’t respond to anti-seizure medications (Wood 2019).

Bioelectronic medicine could improve our understanding of the brain and the biological mechanisms underpinning such disorders – which could lead to new treatments that improve health outcomes and reduce costs. For example, Neuronetics has already provided more than 2 million treatments for major depressive disorder with its technology, called transcranial magnetic stimulation (TMS) (Neuronetics 2018). Deep brain stimulation (DBS) has also shown promise for depression treatment; in one recent study, DBS achieved a more than 40% drop in depression score for 50% of patients, and remission for 31%, when used for 2 years (Dobbs 2018). There are also a number of approved bioelectronic medicine treatments for conditions like epilepsy, Parkinson’s, and movement disorders (Tsao 2018).

#### Chronic condition prevention and management

When used together with existing tools for diagnosis and treatment, bioelectronic medicine could have applications for the prevention, screening, treatment, and care of “modern epidemics” – the pervasive chronic conditions like diabetes and obesity that affect many millions of people around the world.

In some cases, bioelectronic medicine has been shown to be an effective tool for the early detection of these chronic diseases. For example, clinical research from AliveCor – a company focused on collecting and analyzing patients’ heart rhythm data – has demonstrated the feasibility of analyzing ECGs to improve early diagnosis of atrial fibrillation and then take action to prevent stroke (AliveCor 2019). Further, efforts like Verily’s Project Baseline and the Apple Heart Study show that many people are eager to participate in high-tech research and screening efforts (Verily 2019; Stanford Medicine 2018).

Bioelectronic medicine could tap into this enthusiasm and enable individuals to see their own bioelectronic data and how their bodies respond to lifestyle changes and treatment on a real-time, ongoing basis. This capability could drive patient empowerment, health literacy, and disease prevention and management.

Finally, bioelectronic medicine also has the potential to directly fight chronic conditions. For example, early research at the Feinstein Institute and GE has shown that noninvasive ultrasound can be used to regulate the body’s metabolic and inflammatory control systems and modulate blood glucose levels, with potential applications for diabetes, arthritis, inflammatory bowel disease, and other diseases. (The Feinstein Institute for Medical Research 2019; GE Research 2019).

#### Meeting the global need for healthcare

Bioelectronic medicine could play a role in helping to address the urgent, growing shortage of health workers globally, especially in low- and middle-income countries (LMICs). The World Health Organization reports that 400 million people do not have access to essential health services (World Health Organization 2015), and experts project that LMICs’ unmet need for health workers will more than double from 6.6 million in 2013 to 15.5 million in 2030 (Lui et al. 2017).

Bioelectronic medicine has produced some early examples of lower-cost tools that could help to improve global access to health information, diagnosis, and potentially treatment. For example, the D-EYE Smartphone-Based Retinal Imaging System (D-EYE) is an affordable attachment and companion application for a smartphone that enables early diagnosis of eye diseases (D-EYE n.d.). D-EYE has already expanded access to eye health in Asia and South America, and its creators plan to work towards applications for other diseases.

Future bioelectronic medicine applications could follow a similar path, combining affordable tools and advanced analytics to drive better access to healthcare globally.

#### Opportunities for artificial intelligence

When integrated with artificial intelligence (AI), bioelectronic medicine opens new opportunities for improved diagnosis, predictive insights, and more effective, personalized treatments. These technologies are capturing a growing array of biological signals – from ECG and EEG to breath and voice – that can provide the basis for AI to inform and advance healthcare. By analyzing these huge datasets, research projects have shown that AI can help to drive earlier diagnosis, including for patients that appear to be asymptomatic.

For example, the cardiovascular AI team at the Mayo Clinic used tens of thousands of ECG pairs to train a neural network to identify those with heart failure. The network was then able to identify heart failure with a high degree of accuracy (Mayo Clinic 2019). In addition, the network was able to detect risk for developing future heart problems, suggesting that it may be able to reveal abnormalities hidden in the ECG.

In another example, researchers with Google DeepMind and the U.K.’s Moorfields Eye Hospital showed that their AI system can interpret eye scans to diagnose more than 50 eye diseases with the same level of accuracy as human experts (Suleyman 2018). This AI system also provides a description of the symptoms it detects and a percentage certainty of its diagnosis, so that providers can understand the reasoning for a diagnosis.

There are opportunities to develop such AI-based diagnostics for other disease areas and integrate these tools into real-world practice. AI may also have a role to play in developing novel therapies and continuously optimizing those therapies.

## Defining a shared vision for bioelectronic medicine

Bioelectronic medicine has reached a critical stage in its development. To achieve its full promise, those working within the field must join together to paint a compelling vision of the future: What exactly is bioelectronic medicine? Why should we be excited about it? How are these innovations destined to advance healthcare diagnostics and treatment? Crafting and communicating this shared vision will be key to driving wider engagement and support from external audiences, including physicians and providers, patients, policymakers and regulators, payers, investors, and society.

Breakthroughs in bioelectronic medicine are being achieved today by researchers working in a largely siloed manner across multiple disciplines and disease areas. The good news is that we have seen important progress in recent years towards greater knowledge-sharing and collaboration, including a number of convenings, the creation of a new medical journal, and the publication of consensus statements and research roadmaps (Center for Molecular Medicine 2018; Tyler et al. 2017; Bioelectronic Medicine 2019; Pavlov and Tracey 2019). Also worth noting is the NIH Common Fund’s Stimulating Peripheral Activity to Relieve Conditions (SPARC) program, which aims to establish effective research partnerships with clinicians, basic scientists, engineers, and private industry, and has created a public portal for sharing data and resources (National Institutes of Health 2020). However, it remains difficult for early movers to stay abreast of the latest developments outside their own specialty area or to collaborate broadly.

Moreover, public awareness of bioelectronic medicine remains relatively low, and little is being done to educate or engage external audiences. Some important audiences – such as investors and older patients – are largely unaware of bioelectronic medicine and its potential benefits. Others – such as physicians and payers – are more aware, yet skeptical of some claims or hesitant to make real-world changes.

### Catalysts for advancing bioelectronic medicine

Against this backdrop, we see three catalysts for advancing bioelectronic medicine as an emerging industry worthy of investment and excitement: a shared definition, a unified narrative, and a set of tailored messages for key audiences.

#### **Catalyst #1:** a shared definition that is both inclusive and meaningful

Currently, the terms and definitions used to describe bioelectronic medicine often focus too narrowly on one discipline (e.g., neuromodulation) and are not cohesive. The field needs a single, widely used definition of “bioelectronic medicine” that reflects the broad range of disciplines, technologies, and organizations contributing to its development. However, this definition also needs to filter out the significant noise in related areas, including less reputable technologies.

- **Proposed definition:** Bioelectronic medicine explores how targeted electrical signals can harness the body’s natural mechanisms to diagnose and treat a range of diseases.

#### **Catalyst #2:** a unified narrative

To date, there have been a number of conferences and collaborative initiatives exploring technical and scientific topics related to bioelectronic medicine. Still, there is an outstanding need for an overarching narrative and messages that clearly articulate how bioelectronic medicine stands to benefit people, including its unique advantages and potential for growth.

We propose the following messages as the most compelling way to describe bioelectronic medicine and differentiate it from other, more familiar healthcare practices. These messages must reflect the differences between diagnostics and therapeutics: it will likely be easier to build initial acceptance of diagnostics, while therapeutics may face more skepticism, given the potential for adverse effects.

##### Diagnostics and data

- **Better diagnosis and prevention.** Bioelectronic medicine can guide earlier, more accurate diagnosis, which can then be validated by a healthcare provider. These capabilities can enable more effective, less costly prevention and intervention.
- **Personalized data.** Bioelectronic medicine can collect personalized health data – in real time and continuously – from a wide variety of body systems. Such a large amount of connected data opens unprecedented new opportunities for analytics to improve healthcare.
- **Patient empowerment and physician support.** Access to personalized bioelectronic data can facilitate greater patient understanding of their conditions and greater engagement with their treatments, higher levels of health literacy, and greater communication and trust between patients and physicians.

##### Therapeutics

- **Tailored to the individual.** With the ability to both sense and intervene, bioelectronic medicine treatments can be closely tailored to the needs of an individual – responding on demand to the core causes of a disease, not just its symptoms.
- **Real-time, continuous, and optimized.** Bioelectronic medicine treatments can provide care that is both real-time and continuous – intervening in response to specific changes and optimizing the treatment based on feedback loops.
- **Targeted, natural mechanisms.** Bioelectronic medicine taps into the body’s own homeostasis mechanism to target specific causes of disease, which can avoid the need for invasive medical procedures or use of drugs with adverse effects.

#### **Catalyst #3:** tailored messages for key audiences

**Fig. 2:**
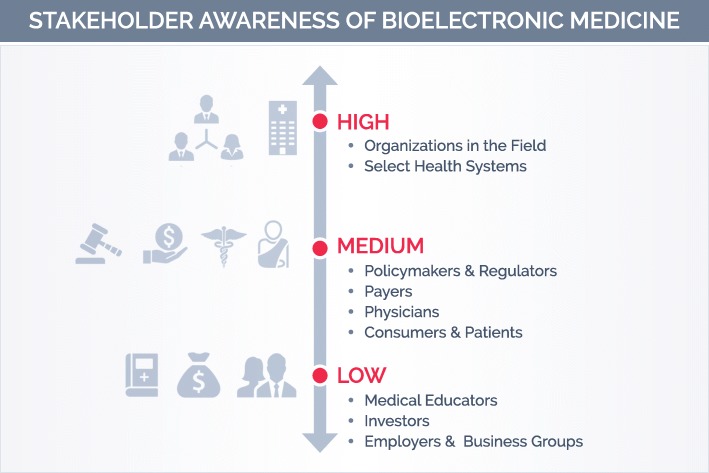
Stakeholder Awareness of Bioelectronic Medicine

The future of bioelectronic medicine will depend on building awareness and converting skeptics to supporters. Figure 2 summarizes our assessment of current levels of awareness among different stakeholder groups.

To raise awareness, the field needs tailored messages that acknowledge the needs and concerns of specific external audiences and zero in on relevant benefits. For each stakeholder group, we need to explain how bioelectronic medicine can help achieve their goals, while addressing their concerns about potential challenges. The landscape analysis below explores the opportunities and challenges for engaging seven key external audiences:

- Physicians
- Consumers and patients
- Policymakers and regulators
- Payers
- Investors
- Medical educators
- Employers and business groups

##### Physicians

Broadly speaking, physicians have a medium level of awareness of bioelectronic medicine. However, many physicians are skeptical due to the wide range of claims from some in the field. They will require more high-quality evidence of efficacy before accepting and adopting these applications for diagnosis and, especially, for treatment.

Physicians also have concerns about how the massive amounts of data available from bioelectronic medicine applications will impact their workload. Many physicians don’t want to have to interpret new troves of data, as has already been seen with some ECG technologies. Moreover, they also don’t trust “black box,” AI-based applications that they can’t validate – i.e., situations where AI may generate a decision or diagnosis without being able to fully explain the underlying factors or the certainty of the decision.

**Effective engagement with physicians will:**

- **Build proof of efficacy.** The key to physician acceptance is high-quality evidence that bioelectronic medicine is accurate and effective, and that it provides added value over existing tools. Stakeholders in the field must focus more attention and investment on generating this evidence, particularly through clinical trials and publication in respected medical journals and, in a later phase, through engagement with payers.
- **Demonstrate improved physician workflow.** Physicians would be more accepting of bioelectronic medicine if it was shown that these tools could reduce their workloads by improving workflow, aiding triage, and enabling nurses or other healthcare providers to take on greater responsibilities.
- **Develop mechanisms for AI to address uncertainty and false positive/negatives.** Bioelectronic medicine stakeholders will need to work with physicians and other stakeholders to define thresholds for error in AI-based diagnostics and decisions. Color-coded ranks for certainty, like those used in remote care, could be one approach.
- **Start with forward-looking key opinion leaders (KOLs) and health systems.** A growing number of renowned physicians and health systems – such as Mount Sinai, Mercyhealth, Cleveland Clinic, and others – are more interested and accepting of bioelectronic medicine. These health systems are already piloting bioelectronic medicine devices with certain patient populations, and they represent an important opportunity to build trust, demonstrate efficacy, and serve as a model for other healthcare systems.

##### Consumers and patients

Overall, consumers and patients have a medium awareness of bioelectronic medicine. However, awareness varies significantly and is highest among patients and caregivers in disease areas that lack effective treatments, as well as among younger individuals. Groups with higher levels of awareness are eager for new, tech-based medical solutions – and surprised that they don’t already exist or aren’t widely available.

**Effective engagement with consumers and patients will:**

- **Build broad public awareness, not just with patients.** Consumers will be able to access some bioelectronic medicine applications without a prescription – suggesting the need (and the opportunity) to spark interest among individuals *before* they become patients.
- **Engage patients in the development of new technologies.** Integrating patients into the design process could lead to bioelectronic medicine applications that better meet patients’ needs, offer desired features, and build demand for and acceptance of novel diagnostic and therapeutic solutions.
- **Connect patients to influence other stakeholders.** Patients and advocacy groups in certain disease areas are eager for new healthcare approaches, and their voices should weigh in the decisions of regulators, policymakers, and payers. By engaging these advocates, bioelectronic medicine stakeholders can make a stronger case for the field.

##### Policymakers and regulators

Regulators and other health policymakers are generally aware of bioelectronic medicine, but policy hurdles and skepticism vary significantly across geographies. The experience of bioelectronic medicine pioneers suggests that regulatory and bureaucratic barriers are higher in the U.S. than in Europe. Globally, there are examples of national health systems that are starting to more fully explore innovative applications, such as the integration of AI in Singapore or the use of biometric ID cards in India.

AI, in particular, presents significant challenges for regulators, who are already struggling to define frameworks that can accommodate the exploding size and complexity of health data being produced these days. Much like physicians, regulators are uncomfortable with “black box” AI systems that cannot explain their decisions.

**Effective engagement with regulators will:**

- **Amplify patient voices.** In both the U.S. and Europe, patient preferences are increasingly factored into health policy and regulatory decisions. This provides an opportunity for bioelectronic medicine players to engage with patients and advocacy groups in certain disease areas to amplify their calls for new treatment approaches.
- **Focus on disease areas and applications with lower regulatory hurdles.** Regulators assess treatments based on the level of unmet need in the disease area, the severity of the condition, and the potential for harm. Therefore, bioelectronic medicine treatments that target high levels of unmet need and severe conditions, such as major depressive disorder, may face a lower bar for approval.
- **Develop methods to help explain the “black box” of AI.** Providing a general description of the most critical factors that influence an AI-based decision, without fully explaining every factor, could help to ease regulators’ concerns in this area. Additionally, AI-based decisions could include an estimate of the system’s degree of certainty.

##### Payers

Payers’ awareness and interest in bioelectronic medicine varies across geographies. Participants at the roundtable noted that payers in the U.S. and Israel have added billing codes for bioelectronic medicine, indicating their willingness to experiment to potentially realize cost savings. However, European payers appear to be more conservative and less interested.

**Effective engagement with payers will:**

- **Harness evidence of cost-effectiveness in clinical trials.** Payers could drive adoption of bioelectronic medicine if they define it as high-quality care in clinical standards. However, payers will likely require evidence-based validation of innovation and cost effectiveness to take this step.

##### Investors

Many in the investment community are not aware of bioelectronic medicine. However, recent IPOs such as those from Inspire Medical, Axonics Modulation Technologies, electroCore, and Neuronetics are beginning to spark interest in the field’s growth potential. Indeed, some of these companies have now achieved market capitalizations approaching or surpassing $1 billion (Yahoo! Finance 2019a, 2019b). Investors would likely take note if they started hearing a unified narrative from those in the field – either directly from innovators or via the media.

**Effective engagement with investors will:**

- **Connect investors with physicians and companies.** Many physicians and companies in the field want help connecting with investors, and certain investors would be eager to connect – if they were aware of the field’s potential. This creates an opportunity for new forums and mechanisms to connect these two groups for their mutual benefit.
- **Encourage investment in clinical trials.** Investors who are aware of bioelectronic medicine often don’t account for the cost of clinical trials. Engagement and messaging with investors should highlight the benefits of robust clinical trials and resulting evidence, which is necessary to win physician, payer, and regulatory support.
- **Direct public and philanthropic funding to research in the field.** Government research organizations, like the National Institutes of Health, can fund basic, pre-clinical, and clinical research in bioelectronic medicine, helping to address the investment gap at these stages. Philanthropic foundations, especially those focused on disease areas with potential bioelectronic medicine applications, may also be willing to fund relevant research.

##### Other potential audiences

In addition to the key audiences above, participants at the roundtable also mentioned medical educators and employers and business groups as stakeholders that could support the growth of bioelectronic medicine:

- **Medical schools, continuing medical education (CME) providers, and other educators can play a vital role in promoting bioelectronic medicine and early adoption of new approaches.** Certain medical schools are beginning to incorporate engineering into their curriculum and instruction, providing an opportunity to promote bioelectronic medicine. In addition, medical professional societies can educate physicians on new bioelectronic medicine innovations through CME offerings.
- **Employers and business groups have a strong interest in lowering healthcare costs.** If bioelectronic medicine diagnostics or treatments are proven to reduce costs relative to existing tests or pharmaceuticals, these stakeholders would have a vested financial interest in piloting and scaling the use of these applications. This is especially relevant given the growing corporate focus on employee wellness. If ubiquitous and unobtrusive bioelectronic technology can contribute to this goal, companies could become strong advocates for the field.

## Building a bioelectronic medicine movement

Bioelectronic medicine is making rapid strides, but this is just the beginning of what’s possible. If supported by robust investment, appropriate health policy, regulation, and reimbursement, and widespread physician and patient adoption, this field could address some of the greatest challenges in healthcare: from long-standing areas of unmet need to soaring costs to the rising prevalence of chronic conditions.

By taking the following specific actions to advance the field, diverse stakeholders – both those working in the field today as well as external influencers – can help to build a true bioelectronic medicine movement. Ultimately, this engagement and support will be just as important for bioelectronic medicine’s future as scientific and technical progress.

### The bioelectronic medicine field

#### Speak in unison to strengthen the entire field

The diverse leaders and organizations working in bioelectronic medicine today can collaborate to build a unifying identity and vision that encompasses the entire field. Working towards a shared set of messages, policy goals, and public engagement strategies, and speaking with one voice would likely yield heightened awareness and support that benefits bioelectronic medicine as a whole.

#### Build the case for external audiences

Bioelectronic medicine stakeholders can – and must – develop the evidence to persuade external audiences that these applications are safe, effective, cost-effective, and practical. This responsibility reaches beyond regulatory approval, as the field must build a compelling case for why patients, physicians, health systems, and payers should embrace and use these applications in the real world.

#### Collaborate across disciplines to improve understanding of fundamental mechanisms

Those in the field can work across disciplines to advance the foundational science of bioelectronic medicine. This will include research on molecular targets, mechanisms of action, biomarkers, phenotypes, and other topics, drawing on interdisciplinary expertise and collaboration. This will be essential not just to improve diagnostics and treatments, but to win greater acceptance with external audiences.

#### Provide high-quality evidence of efficacy, cost effectiveness, and comparisons with existing treatments

The bioelectronic medicine field can conduct clinical and post- market research to generate evidence of whether and how bioelectronic medicine benefits patients, improves health outcomes, and reduces costs. This research will need to include comparisons with existing treatments and information on adverse events. And for credibility, it must meet the publication standards of well-regarded journals.

### Physicians

#### Partner to conduct clinical research and pilot programs

Forward-looking physicians can work with bioelectronic medicine stakeholders to conduct clinical trials and pilot programs that examine treatment efficacy and cost effectiveness. In this role, physicians can provide important guidance to ensure research results will be credible and accepted. Medical societies that publish research-based practice guidelines can include proven bioelectronic diagnostic and therapeutic tools in their recommendations.

#### Include sessions and content on bioelectronic medicine at medical society convenings

Bioelectronic medicine is emerging as an innovative and increasingly important frontier in many specialties, from ophthalmology to gastroenterology. Special programming at existing medical society convenings can spotlight these innovations, educate practitioners, and build wider acceptance.

#### Connect with engineers

By connecting with engineers for knowledge-sharing and collaboration, physicians can stimulate cross-pollination that informs and improves research, clinical trials, and uptake of bioelectronic medicine applications.

### Consumers and patients

#### Seek reputable sources of information on bioelectronic medicine

An informed public that understands the potential of bioelectronic medicine can act as the field’s greatest ally. Media coverage of innovative solutions and KOLs will play a large role in shaping public awareness and attitudes – and, to do their jobs well, journalists must have easy access to credible, unbiased information. To that end, leaders in the bioelectronic medicine space will need to use public speaking and media opportunities to advance the entire field, not just to promote their own interests.

#### Call on regulators, policymakers, and payers to explore bioelectronic medicine

Patient advocacy groups, especially those in areas with few effective treatments, can act as key voices of support for bioelectronic medicine in regulatory, reimbursement, and policy decisions.

### Policymakers and regulators

#### Consider the implications and unique advantages of bioelectronic medicine for current and future health policy and regulation at the global, national, and community levels

For example, global health policymakers could showcase the opportunity for affordable diagnostic tools, while national campaigns to fight chronic conditions could include bioelectronic screening tools.

#### Provide guidance to help navigate regulatory pathways

Regulators can create designated groups and experts to help bioelectronic medicine researchers and companies understand and meet requirements for new devices. Such guidance would be particularly valuable for start-ups with limited resources that are bringing innovative devices to market for the first time.

#### Develop regulatory frameworks for AI

To enable AI-based innovation, regulators can develop frameworks that consider the most important factors in an AI decision or diagnostic, without requiring an explanation of every factor.

### Payers

#### Introduce billing codes for bioelectronic medicine

Payers can explore the potential for cost savings from bioelectronic medicine by introducing billing codes that incentivize the use of these applications for diagnosis, monitoring, and treatment.

#### Analyze the cost savings of bioelectronic medicine

After introducing billing codes, payers can analyze real-world results to understand and quantify the potential cost savings of bioelectronic medicine for certain uses and patient populations.

#### Consider integrating these applications into clinical care guidelines

If bioelectronic medicine applications are shown to reduce costs and improve outcomes, payers can integrate them into clinical care guidelines to drive adoption by providers.

### Investors

#### Connect with companies and physicians in the field

Bioelectronic medicine companies and innovators are eager for funding, and many investors would likely be interested in the field – if they knew about it. This presents an important opportunity for mutually beneficial connection and collaboration.

#### Account for the cost of clinical trials

Investors must anticipate the need to fund clinical trials that can produce high-quality evidence of bioelectronic medicine’s treatment efficacy and cost effectiveness. This is the only way to win over skeptical audiences and drive widespread adoption.

### Educators

#### Integrate engineering into medical education

Medical schools can integrate engineering into their curriculum, hire instructors who have engineering backgrounds, and require students to complete bioengineering projects. Medical training should include bioelectronic diagnostic and therapeutic tools as part of the education for all students.

#### Include bioelectronic medicine in continuing medical education

Organizations that provide continuing medical education (CME) can showcase bioelectronic medicine applications as an area of important innovation. This is especially relevant for “older” audiences, who may not have had exposure to this field during their training.

### Employers and business groups

#### Pilot bioelectronic medicine applications

There may be an opportunity for large employers and business groups to collaborate with payers in piloting selected bioelectronic medicine applications and analyzing the potential for improved employee wellness and cost savings.

## Conclusion

Bioelectronic medicine is at a tipping point. In recent years, companies have expanded the field’s applications, conducted promising clinical trials, and begun offering treatments to patients. This lays the foundation for important advances in the future.

However, few outside of the field are actively working towards these opportunities. Winning their support will require many diverse leaders and organizations to speak in unison, advancing a single, compelling vision for how bioelectronic medicine can improve people’s lives. In particular, the field needs to advance understanding of fundamental mechanisms and develop and publish clear evidence of benefits for patients, physicians, health systems, and others.

Engaging a wide variety of stakeholders and winning their support is just as important for bioelectronic medicine’s future as scientific and technical milestones. Organizations and leaders need to join together, across sectors and geographies, to create an environment that facilitates the growth of the field and the realization of its potential benefits. We hope that the roundtable, and the resulting insights and collaboration through the Alliance for Advancing Bioelectronic Medicine, will help to guide progress towards these goals.

## Data Availability

Not applicable.

## Abbreviations

AI: Artificial intelligence
CME: Continuing medical education
DBS: Deep Brain Stimulation
LMIC: Low- and middle-income country
NIH: National Institutes of Health
SPARC: Stimulating Peripheral Activity to Relieve Conditions
TMS: Transcranial magnetic stimulation
